# Deep Learning-Based Medical Data Association Rules to Explore the Connectivity and Regulation Mechanism of miRNA-mRNA Network in Myocarditis

**DOI:** 10.1155/2022/9272709

**Published:** 2022-09-23

**Authors:** Fang Li, Jingzhe Li, Jie Hao, Jinming Liu, XiuGuang Zu, Bin Wang

**Affiliations:** ^1^Third Division Department of Cardiology, The Second Hospital of Hebei Medical University, Shijiazhuang, Hebei 050000, China; ^2^Clinical Medicine, Hebei Medical University, Shijiazhuang, Hebei 050017, China

## Abstract

Acute, chronic myocarditis as myocardial localized or diffuse inflammation lesions is usually involving cardiac function in patients with severe adverse outcomes such as heart failure, sudden death, and no unified, but its pathogenesis clinical is mainly composed of a number of factors including infection and autoimmune defects, such as physical and chemical factors; therefore, it is of great significance to explore the regulation mechanism of myocarditis-related miRNA network connectivity and temperament for in-depth understanding of the pathogenesis of myocarditis and the direction of targeted therapy. Based on this, this study explored the miRNA network related to the pathogenesis of myocarditis through deep learning medical data association rules and analyzed its specific mechanism. The results showed that 39 upregulated miRNAs, 88 downregulated miRNAs, 109 upregulated differentially expressed miRNAs, and 589 downregulated mRNAs were obtained by data association through GSE126677 and GSE4172 databases. GO enrichment and KRGG enrichment analysis showed that the differentially expressed mRNAs were involved in the regulation of a variety of biological processes, cellular components, and molecular functions. At the same time, the miRNA with differentially expressed miRNAs and their corresponding mRNAs were connected to further clarify the specific molecular mechanism of the pathological changes of myocarditis by constructing miRNA-mRNA network. It provides effective potential molecular targets for subsequent treatment and diagnosis.

## 1. Introduction

As we know, the myocardium plays a role in supporting the normal work of the heart in the human body, but when it has a slight lesion, the patient's body may not feel well, and the heart function of patients with severe myocarditis will be seriously impaired, resulting in very serious consequences, such as heart failure and sudden death [1]. At present, the incidence of myocarditis is higher in winter and spring, there are different degrees of incidence probability in all age groups, and it is more common in young and middle-aged people who are usually healthy and without basic structural lesions. At the same time, there is no significant gender difference in the incidence of myocarditis, but the incidence of myocarditis is higher in long-term fatigue group [2, 3]. At present, there is no unified conclusion on the pathogenesis of myocarditis in clinical practice, but the most common cause of myocarditis is viral infection. Second, different bacterial and fungal infections can also cause myocarditis to a certain extent, but its incidence is lower than viral infection [4]. In addition, some relevant studies have pointed out that some noninfectious factors can also cause myocarditis, such as drugs, vasculitis, and radiation [5]. The clinical manifestations and symptoms of myocarditis depend on the severity of the disease. Mild cases may have no conscious symptoms, and severe cases may even have cardiogenic shock, heart failure, severe arrhythmia, sudden death, etc. Therefore, exploring the relevant molecular mechanism of myocarditis plays an important role in preventing and organizing the progression of myocarditis [6].

At present, with the development of gene work, the maturity of gene chip, and sequencing technology, a large amount of biological big data has been generated, which enables people's understanding of diseases to go far from the traditional pathology to the gene level and promotes the birth and development of targeted medicine [7]. Medical data are mainly based on the deep learning gene expression database (GEO) expression of disease spectrum chips on the basis of the data in the data analysis, and by using bioinformatics methods for diseases of differentially expressed genes with the core, through the construction of protein interaction network so as to explore the pathogenesis of disease [8, 9]. At the same time, with the deepening of research, the concept of miRNA has been proposed and confirmed accordingly. As a class of noncoding RNA in cells, miRNA induces mRNA degradation or inhibits mRNA translation of its target gene by completely or incomplete binding to the 3′-untranslated region of its target gene in vivo, so as to achieve the purpose of inhibiting the expression of its target gene [10]. Current clinical research suggests the miRNA by inhibiting the expression of target genes in the body to participate in the cells of a variety of biological actions, including maintaining stem cell function, cell growth, and apoptosis; and a growing number of studies have confirmed the miRNA can as oncogenes or tumor suppressor gene in the process of cancer development play an important role in regulation; therefore, miRNA can be used as an effective biomarker for early diagnosis, prognosis evaluation, and therapeutic effect prediction of a variety of diseases and has a wide application prospect [11].

In order to further clarify the regulatory role and mechanism of miRNAs in myocarditis, it is important to identify the differentially expressed miRNAs in myocarditis and identify their target genes. However, there are relatively few studies to analyze the relationship between miRNA-mRNA at the genome-wide level in myocarditis. Therefore, this study clarified the regulatory relationship of miRNA-mRNA in myocarditis by using in-depth medical data association rules such as gene chip and bioinformatics and did not analyze its possible molecular regulatory mechanism.

## 2. Data and Methods

### 2.1. Data Sources

The microarray dataset of gene expression profile of human myocarditis was searched in the GEO database. The mRNA data was obtained from GSE126677, and myocardial tissue samples from 10 myocarditis patients and 5 healthy people were selected. The miRNA was derived from the RNA sequencing data GSE4172 in the GEO database, including myocardial tissue samples from 8 myocarditis patients and 4 healthy people. The RNA was obtained from the miRNA and analyzed by gene expression profile. Affymetrix Human Genome U133 Plus 2.0 Array was used to analyze and process the gene expression profile of myocarditis, and the corresponding background correction and standardization was performed.

### 2.2. Methods

The differentially expressed miRNAs in myocarditis patients were analyzed by R4.0.3, and the acquired miRNA and mRNA data were standardized by Limma loading package. The differentially expressed miRNAs were obtained at *P* < 0.05, and PhestMap in R software was used to draw the required heat map. Differentially expressed miRNAs between myocarditis patients and healthy people were visualized to establish a protein-protein interaction network.

The functional enrichment analysis of differentially expressed mRNAs was annotated by biological process (BP), cell component (CC), and molecular function (MF), and the enrichment pathway of differentially expressed genes was mainly conducted by DAVID. Fun Rich3.1.3 was used to predict the target genes of differentially expressed miRNAs. The miRNA-mRNA interaction relationship was visualized by drawing Venn diagram.

### 2.3. Statistical Treatment

Data were analyzed by R 4.0.3, and Wilcox test was performed. Gene differential expression was analyzed by *t* test. *P* < 0.05 was considered statistically significant.

## 3. Results

### 3.1. Screening Differentially Expressed mRNAs and miRNAs

**Figure 1:**
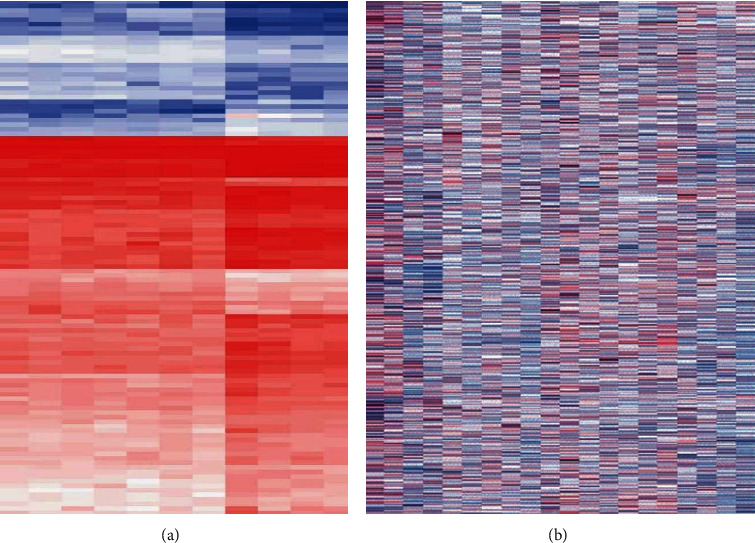
Heat map of differential expression of mRNA and miRNA.

**Figure 2:**
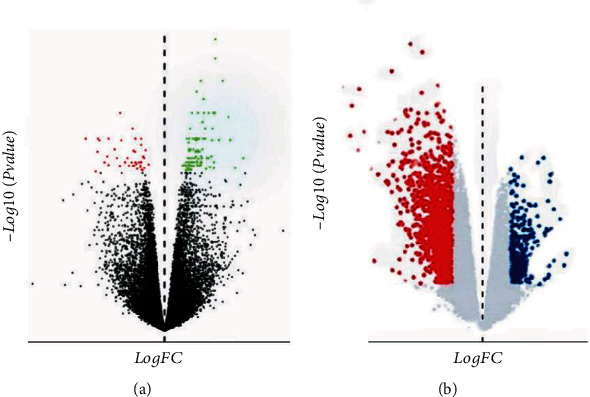
Volcano map of miRNA and mRNA differential expression. Note: (a) is the volcano map corresponding to GSE4172 database, and (b) is the volcano map corresponding to GSE126677 database. In (a) and (b), red showed high expression and blue showed low expression.

The results of differential gene expression analysis showed that a total of 127 miRNAs were differentially expressed in myocarditis tissues in the GSE4172 database, of which 39 were upregulated and 88 were downregulated, as shown in Figure 1(a). The GSE126677 database showed that there were 698 differentially expressed mRNAs in myocarditis tissues, of which 109 were upregulated and 589 were downregulated, as shown in Figure 1(b). The volcano plots of differential gene expression profiles were shown in Figures 2(a) and 2(b), respectively.

### 3.2. Construction of mRNA PPI Network and Identification of Core Genes

**Figure 3:**
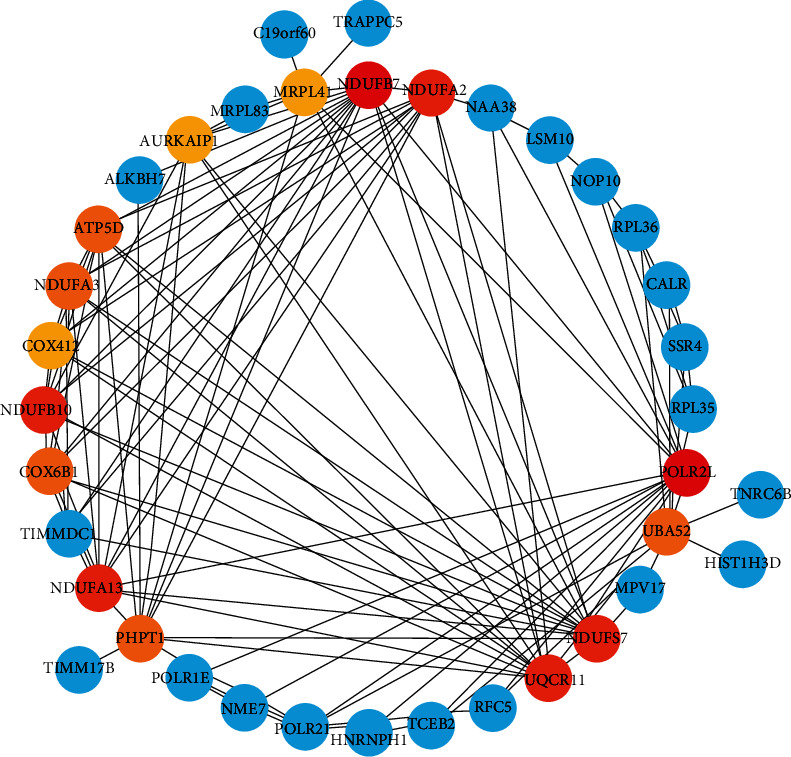
PPI network constructed by differential miRNAs.

The constructed PPI visual network is shown in Figure 3, and a total of 13 core genes ranked higher were screened out. The specific test data are shown in Table 1. The darker the color, the higher the degree value.

### 3.3. Enrichment Analysis of Differentially Expressed miRNA GO

**Figure 4:**
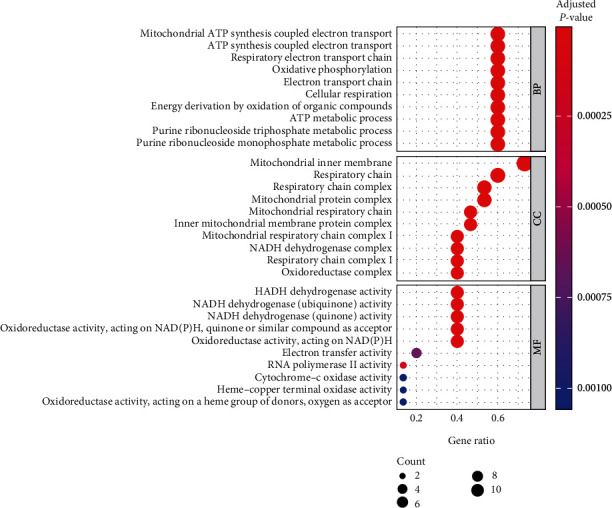
GO enrichment analysis bubble plot of miRNA differential expression.

GO analysis showed that the biological processes mainly included mitochondrial ATP synthesis coupled electron transport, respiratory electron transport chain, oxidative phosphorylation process, cellular respiration process, and oxidative energy generation of organic compounds. Cell components include NADH dehydrogenase, mitochondrial respiratory chain complex I, and mitochondrial protein complex. Molecular functions mainly include oxidoreductase activity and NAD(P) H, RNA polymerase II activity, and cytochrome oxidase activity, as shown in Figure 4.

### 3.4. KEGG Enrichment Analysis

**Figure 5:**
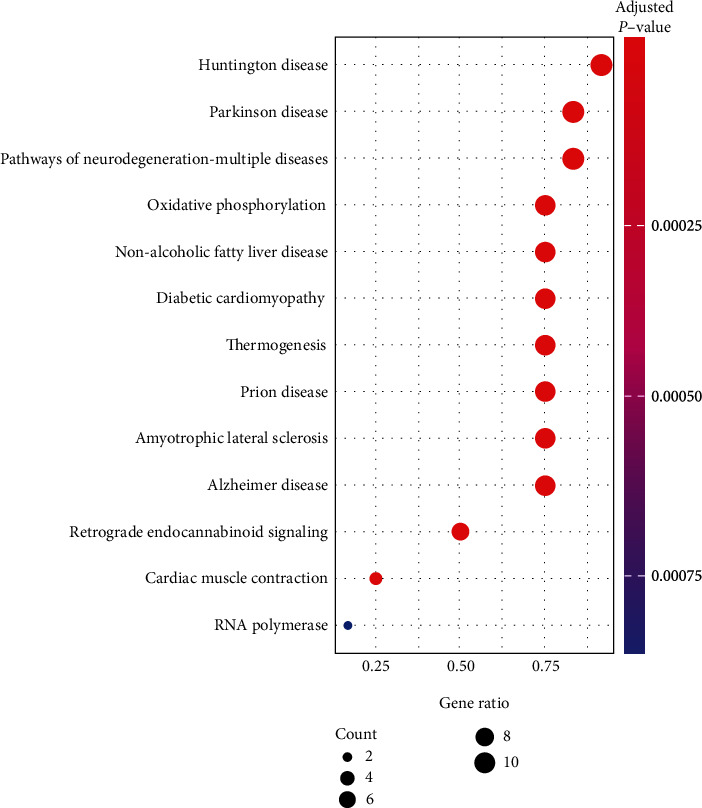
KEGG enrichment analysis bubble diagram.

The results of KEGG enrichment of miRNAs with major differences showed that they were mainly concentrated in 13 aspects, including oxidative phosphorylation, Parkinson's disease, nonalcoholic fatty liver disease, diabetic cardiomyopathy, and myocardial contraction, as shown in Table 2 and Figure 5.

### 3.5. Construction of miRNA-mRNA Regulatory Network

**Figure 6:**
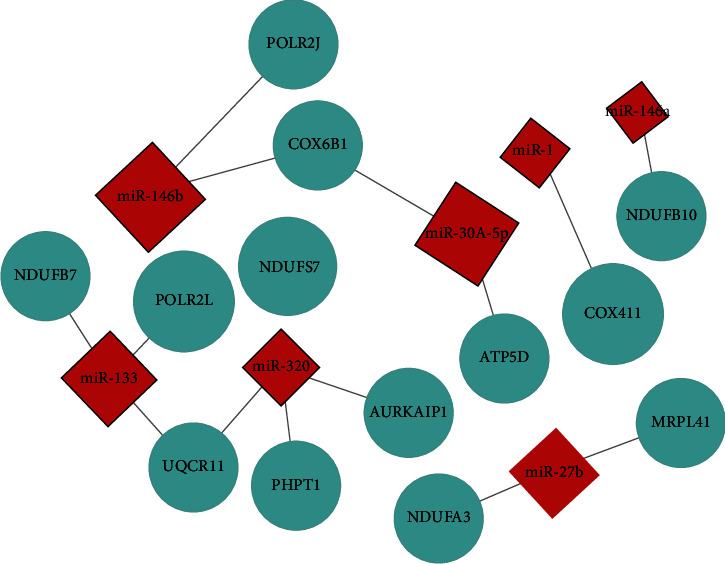
miRNA-mRNA interaction network. Note: in the figure, red represents upregulation and blue represents downregulation.

The predicted target genes should be intersected with the downregulated differentially expressed mRNAs. A total of 13 downregulated mRNAs were obtained by drawing the Venn diagram. The expressions of related miRNAs and mRNAs are shown in Table 3, and the specific network diagram is shown in Figure 6.

## 4. Conclusion

Myocarditis patients according to clinical research of microRNAs can be characterized by specificity raised or lowered, with myocarditis has very close connection between onset, age is no related research illustrate myocarditis specific mechanism of the disease, but can be based on the analysis of microRNAs and mRNA expression differences on the possible molecular mechanism of speculation and provide new therapeutic targets.

### 4.1. The Relationship between miRNA and Myocarditis

It has been found that the upregulation of miR-21 and miR-146b can promote the differentiation of TH17 cells and increase the release of interleukin-17, thereby aggravating myocardial inflammatory response, suggesting that miR-21 and miR-146b are involved in the pathogenesis of viral myocarditis. miR-155 and miR-148a can reduce the expression of interleukin-6 and interleukin-1*β* by inhibiting RelA (p65), a subunit of nuclear transcription factor rB, while miR-146a can play a protective role in myocardium by targeting TLR3 and TRAF6 to block the nuclear transcription factor kB pathway. miR-381 can bind to the 3′-untranslated region of COX-2 mRNA and inhibit its expression, thereby inhibiting the inflammatory response of cardiomyocytes and reducing the damage of cardiomyocytes [12, 13]. Meanwhile, in other studies, miR-34a was highly expressed in CVB3-induced myocarditis cell culture model, which resulted in the downregulation of SIRT1 expression. Other researchers have suggested that miR-217 and miR-543 have the same mechanism. SIRT1 is a core component of the SIRT1-p53 signaling pathway and an important inhibitor of apoptosis, and its downregulation further promotes apoptosis. miR-98 can affect the pathogenesis of myocarditis by binding to FAS/FASL gene targets [14, 15]. Fas is a membrane surface molecule, its ligand FASL can affect a number of apoptosis-inducing signal transduction pathways, and miR-98 can inhibit the expression of Fas/FASL gene in cardiomyocytes by binding to Fas and FASL and then reduce cell apoptosis [16].

### 4.2. The Relationship between mRNA and Myocarditis

GO enrichment analysis in this study showed that the differentially expressed genes were mainly distributed in mitochondrial ATP synthesis coupled electron transport, respiratory electron transport chain, oxidative phosphorylation, respiratory chain, mitochondrial intima, NADH dehydrogenase activity, oxidoreductase activity, etc. Meanwhile, core genes were mainly enriched in oxidative phosphorylation, nonalcoholic fatty liver disease, diabetic cardiomyopathy, myocardial contraction, and other pathways. Among them, NADH is the largest protein complex in oxidative phosphorylation, and homozygous mutation in the intron of its gene will reduce the activity of the complex, thus exhibiting the clinical characteristics of hypertrophic cardiomyopathy. At the same time, Rotllan et al. [17] also pointed out that in the process of ischemia-reperfusion, if the proteolysis of NDUFS7 is increased, the activity of the complex will be reduced to varying degrees, leading to the aggravation of myocardial injury, while NDUFA13 gene knockout has a protective effect on myocardium.

COX6B1 as cytochrome magnesia sixth subcomponents, its main role is in the body by connect two cytochrome magnesia monomer into specific triggering and myocarditis, and relevant research shows COX6B1 to a certain extent can reduce the damage brought by myocardial ischemia, and the mutation can cause cardiomyopathy; however, there are relatively few basic studies on other core genes and cardiomyopathy, so the specific molecular mechanisms need to be confirmed by further studies.

In addition, KEGG analysis showed that core genes were significantly distributed in the oxidative phosphorylation pathway, suggesting that they play an important role in the process of cardiomyocyte energy metabolism. Cardiomyocyte damage is the main pathogenesis of heart failure, and the decline of cardiomyocyte function is closely related to a variety of biological processes. It is closely related to the regulation of calcium ion in cytoplasm and mitochondria, suggesting that improving the regulation of calcium ion in cardiomyocytes may be an effective therapeutic target in the clinical treatment of myocarditis.

In summary, miRNA-mRNA is involved in multiple signaling pathways that are closely related to the occurrence and development of myocarditis. Increasing the expression of miRNA-mRNA may be an effective therapeutic target for the treatment of myocarditis in clinical practice.

## Figures and Tables

**Table 1:** The differentially expressed mRNA data were analyzed.

Hub genes	Log FC	AveExpr	*t*	*P*
NDUFB7	-1.12	15.38	-5.83	0.001
POLR2L	-0.82	14.39	-7.53	0.008
UQCR11	-1.24	16.93	-6.43	0.042
PHPT1	-0.78	15.38	-5.93	<0.001
NDUFA3	-0.92	16.48	-5.33	0.024
AURKAIP1	-1.04	15.39	-5.82	0.019
MRPL41	-0.76	14.24	-6.93	0.005
COX4I1	-1.17	13.87	-7.38	0.027
ATP5D	-0.70	16.73	-5.61	0.031
NDUFB10	-0.87	14.38	-4.80	0.018
NDUFS7	-1.18	15.82	-6.29	<0.001
COX6B1	-0.97	14.34	-5.82	0.018
POLR2J	-0.82	15.73	-6.77	0.007

**Table 2:** KEGG enrichment analysis.

ID	Description	*P*
Hsa00190	Oxidative phosphorylation	<0.001
Hsa05012	Parkinson disease	<0.001
Hsa04932	Nonalcoholic fatty liver disease	<0.001
Hsa05415	Diabetic cardiomyopathy	<0.001
Hsa03020	RNA polymerase	<0.001
Hsa04260	Cardiac muscle contraction	<0.001
Hsa04723	Retrograde endocannabinoid signaling	<0.001
Hsa05010	Alzheimer disease	<0.001
Hsa00172	Huntington disease	<0.001
Hsa05262	Pathways of neurodegeneration-multiple disease	<0.001
Hsa01824	Thermogenesis	<0.001
Hsa03745	Prion disease	<0.001
Hsa04281	Alzheimer disease	<0.001

**Table 3:** miRNA and mRNA expression with potential relationship.

	Name	*P*	Log_2_FC
miRNA	miR-133	<0.001	1.526
	miR-146b	<0.001	1.762
	miR-1	<0.001	2.281
	miR-146a	<0.001	1.927
	miR-27b	<0.001	1.046
	miR-320	<0.001	2.173
	miR-30a-5p	<0.001	6.423

mRNA	NDUFB7	<0.001	-1.126
	POLR2L	<0.001	-0.824
	UQCR11	<0.001	-1.246
	PHPT1	<0.001	-0.784
	NDUFA3	<0.001	-0.922
	AURKAIP1	<0.001	-1.042
	MRPL41	<0.001	-0.764
	COX4I1	<0.001	-1.178
	ATP5D	<0.001	-0.700
	NDUFB10	<0.001	-0.873
	NDUFS7	<0.001	-1.184
	COX6B1	<0.001	-0.973
	POLR2J	<0.001	-0.825

## Data Availability

The dataset used in this paper are available from the corresponding author upon request.
